# *Candidatus* Neoehrlichia mikurensis and *Hepatozoon* sp. in voles (*Microtus* spp.): occurrence and evidence for vertical transmission

**DOI:** 10.1038/s41598-023-28346-0

**Published:** 2023-01-31

**Authors:** Katarzyna Tołkacz, Maciej Kowalec, Mohammed Alsarraf, Maciej Grzybek, Dorota Dwużnik-Szarek, Jerzy M. Behnke, Anna Bajer

**Affiliations:** 1 Department of Eco-epidemiology of Parasitic Diseases, Institute of Developmental Biology and Biomedical Sciences, Faculty of Biology, University of Warsaw, 1 Miecznikowa Street, 02-096 Warsaw, Poland; 2 Institute of Biochemistry and Biophysics, Polish Academy of Sciences, Pawińskiego 5A, 02-106 Warsaw, Poland; 3 Department of Tropical Parasitology, Institute of Maritime and Tropical Medicine in Gdynia, Medical University of Gdansk, Powstania Styczniowego 9, 81-512 Gdynia, Poland; 4 School of Life Sciences, University of Nottingham, University Park, Nottingham, NG7 2RD UK

**Keywords:** Parasitology, Parasitic infection

## Abstract

*Candidatus* Neoehrlichia mikurensis (CNM) and *Hepatozoon* spp. are important vector-borne parasites of humans and animals. CNM is a relatively recently discovered pathogen of humans. *Hepatozoon* are parasites of reptiles, amphibians and mammals, commonly found in rodents and carnivores worldwide. The present study aimed to determine the prevalence of CNM and *Hepatozoon* spp. in three species of *Microtus* and to assess the occurrence of vertical transmission in naturally-infected voles. Molecular techniques were used to detect pathogen DNA in blood and tissue samples of captured voles and their offspring. The prevalence of CNM in the vole community ranged 24–47% depending on *Microtus* species. The DNA of CNM was detected in 21% of pups from three litters of six infected *Microtus* dams (two *Microtus arvalis* and one *M. oeconomus*) and in 3/45 embryos (6.6%) from two litters of eight CNM-infected pregnant females. We detected *Hepatozoon* infection in 14% of *M. arvalis* and 9% of *M. oeconomus* voles. *Hepatozoon* sp. DNA was detected in 48.7% of pups from seven litters (6 *M.* *arvalis* and 1 *M.* *oeconomus*) and in two embryos (14.3%) obtained from one *M. arvalis* litter. The high prevalence of CNM infections in the *Microtus* spp. community may be a result of a relatively high rate of vertical transmission among naturally infected voles. Vertical transmission was also demonstrated for *Hepatozoon* sp. in *M. arvalis* and *M. oeconomus*. Our study underlines the significance of alternative routes of transmission of important vector-borne pathogens.

## Introduction

*Candidatus* Neoehrlichia mikurensis (CNM) is a relatively recently discovered tick-borne pathogen from the family Anaplasmataceae^1–3^, one of the aetiological agents of so called ‘tick-borne fever’^3–5^. Neoehrlichiosis affects mainly immunocompromised individuals and has been diagnosed also in dogs^3,6^. At least eight species of rodents (*Arvicola terrestris, Apodemus agrarius, Apodemus flavicollis, Apodemus sylvaticus, Myodes glareolus, Micromys minutus, Microtus arvalis, Microtus agrestis*) have been recognised as reservoir hosts of CNM in Europe^7–13^, in addition to *Rattus norvegicus*, the latter species in the first report of the competence of rodents as reservoir for these bacteria^1^. In Central Europe, the main vector of CNM is *Ixodes ricinus* with reported prevalence ranging between 0.1–24.3%^14^. In Poland, CNM has been detected in *I. ricinus* ticks from different habitats including city parks/forests and natural forests with generally low prevalence (0.3–2.9%)^15,16^. Furthermore, CNM has been identified in five immunocompetent asymptomatic foresters from North-Eastern Poland^17^. However, data on the reservoir hosts of CNM in the region of Poland is still fragmentary^18^.

In addition to confirmed transmission by ticks^1,8^, there is also evidence for efficient vertical transmission of CNM in different species of rodents from Germany^10^.

Apicomplexan protists of the genus *Hepatozoon* are parasites of reptiles, amphibians and mammals, commonly found in rodents and carnivores worldwide^15,19–26^. As *Hepatozoon* does not affect livestock or humans, the systematics and transmission routes of these parasites are not well recognised, with many novel species/genotypes identified in rodent hosts still waiting for complete valid descriptions^19,24,26^. Only two main species parasitising dogs, *Hepatozoon americanum* and *Hepatzoozon canis*, are well studied^22^. Canine hepatozoonosis caused by *H. canis* is a common infection in dogs, originally reported from the Mediterranean area of Europe, and more recently also from Central Europe. The first cases of *H. canis* infection in Central Europe were recently recorded in dogs in Hungary^27^, Ukraine^28^, the Czech Republic^29^, Poland (Tolkacz, unpublished), and Germany^30^. Imported *H. canis* cases were also recently diagnosed in the United Kingdom^31^.

*Hepatozoon* spp. are vector-borne parasites, transmitted by the ingestion of different arthropods, including fleas (for rodent species) and ticks, for example the brown dog tick *Rhipicephalus sanguineus* for *H. canis*^32–34^. Other routes of transmission are also suspected, including intake of infected prey (i.e. infected rodents hunted by snakes^24^) and vertical transmission. Vertical transmission has been reported for *H. canis* in dogs in Japan^35^. The high prevalence of *H. canis* in free-living carnivores in Central Europe, in absence of the tick vector, *R. sanguineus*, has led to the conclusion of a possibly high efficiency of transplacental *H. canis* transmission in red foxes, grey wolves, and golden jackals^29,36–39^. In Poland, high prevalence of *Hepatozoon* spp. has been recorded in red foxes, but also in common woodland rodents, i.e. bank voles (*Myodes (Clethrionomys) glareolus*)^20,21,40^. *Hepatozoon* infection was detected also by microscopy in our previous study in common voles, *Microtus arvalis*^41^.

The present study aimed 1) to determine the prevalence of CNM and *Hepatozoon* spp. in three species of voles, based on molecular typing of parasites and 2) to assess the occurrence of vertical transmission of these two vector-borne pathogens in naturally-infected voles.

## Methods

### Scheme of experiments

To investigate the occurrence of transplacental transmission, two field-based experiments were carried out. In the first year, we determined the presence of pathogens in embryos dissected from naturally infected females, since this should completely eliminate the possibility of vector-borne transmission to offspring.

In the second year, to eliminate any possibility of contamination of offspring with maternal blood, we sampled pups obtained from captured, pregnant female voles that were ectoparasite free^42,43^.

### Trapping and processing of voles

Voles were live-trapped in the summers of 2013 and 2014, in long-term abandoned fields near Urwitałt (field station of the University of Warsaw), in the Mazury Lake District of North-Eastern Poland (53°48′50.25"N, 21°39′7.17"E). Three species of voles (common vole *Microtus arvalis*, root vole *Microtus oeconomus*, and field vole *Microtus agrestis*) were trapped in different microhabitats extending up gentle hills (greatest elevation 5 m) from two small mid-field ponds. The local terrain provides a sufficient difference in height for a gradation in physical conditions and vegetation: from marshland submerged during rainy periods that are a suitable habitat for the root vole, *M. oeconomus*, to a dryer grassland habitat preferred by *M. arvalis*. Individuals of *M. agrestis* were trapped mostly in the intermediate zones. Voles were trapped using mixed bait comprising fruit (apple), vegetables (carrot and/or cucumber), and grain. Two traps were set every 10 m along transects at dusk for five consecutive nights, and were checked each morning. Unoccupied traps were then closed after the morning inspection to prevent animals entering during daytime, when excessive heat from exposure of traps to direct sunlight might have affected animals detrimentally, and were re-baited and re-set on the following afternoon. Traps were closed also during periods of rainfall. All the captured animals were transported in their traps to the laboratory for inspection.

In 2013, necropsies were carried out following terminal isoflurane (Merck, Darmstadt, Germany) anaesthesia^42,43^. Voles were assigned to three age classes (juveniles, young adults, and adults) based on body weight and nose-to-anus length together with reproductive condition (scrotal, semi-scrotal or non-scrotal for males; lactating, pregnant or receptive for females)^42^. Two thin blood smears were prepared from blood samples taken by the cardiac puncture of each animal trapped in 2013; additionally, 200 $\mu$l of blood were placed in 0.001 M EDTA and frozen for PCR examination ^42^. Identification of the *Microtus* species was performed as described previously^42,43^. Foetuses were isolated from the uteri, washed in sterile water, and frozen at a temperature of − 20°C^42^.

In the summer of 2014, all voles were live-processed under temporary anaesthesia as described in Tołkacz et al.^42^, during which all ectoparasites were removed. A blood sample for blood smears and PCR examination was taken from the tail tip of each animal. Males, non-pregnant females, and juvenile voles were then released near to their trapping points. Females suspected of being pregnant were transferred to the animal house to be kept in vector-free conditions. Each female was placed in an individual clean sterile cage provided with sawdust, nest material, food (fruit, vegetables, and grain), and water ad libitum, where they were kept until parturition and then with their pups. No ectoparasites were noted on these captive voles at any time after initial caging. Pups were kept together with their dams for one month. In the third week of life, the pups were weighed and blood samples were collected from their tail tips. Pups and dams were then released at the trap lines near to where the dams had been originally caught^42,43^.

### Blood collection and DNA extraction

**Figure 1:**
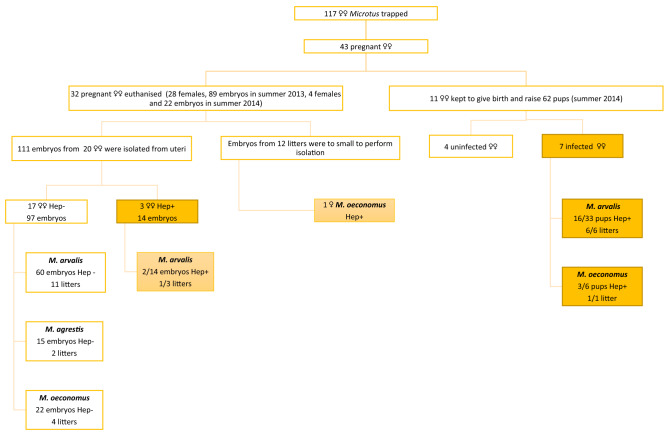
Experimental plan for the study. Hep+ , voles infected with *Hepatozoon* ; Hep− , voles uninfected with *Hepatozoon.*

**Figure 2:**
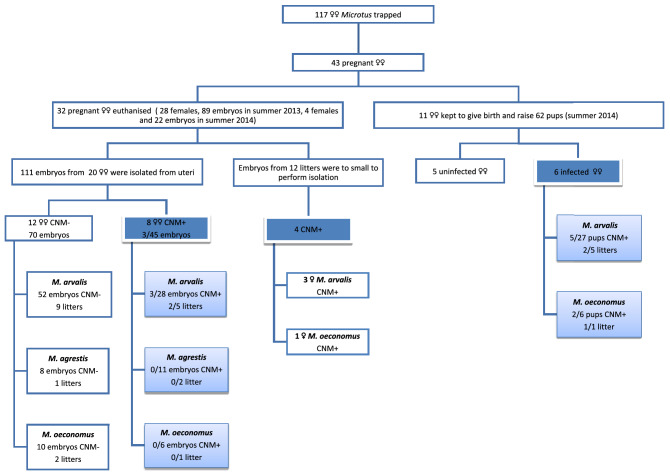
Experimental plan for the study. CNM+ , voles infected with *“Ca.* Neoehrlichia mikurensis*”*; CNM-, uninfected voles.

Embryos were isolated from uteri and individually autopsied following two washes in sterile water, to minimise contamination with maternal blood. We necropsied 111 embryos from 20 litters (Figs. 1, 2). Hearts and lungs were removed from embryos with sterile dissecting instruments. Genomic DNA was extracted from whole blood and organs using the DNAeasy Blood & Tissue kit (Qiagen, NY, USA) and stored at a temperature of − 20 °C. The remaining 12 litters were too small to enable isolation of specific internal organs^43^.

### Microscopic examination

Two blood smears were prepared from trapped voles and pups. Smears were air-dried, fixed in absolute methanol and stained with Diff Quick (Microptic, Barcelona, Spain) or Hemacolor (Merck, Darmstadt, Germany) staining kits, according to the manufacturers' instructions^42^.

Smears from all captured animals and pups were examined for *Hepatozoon* spp. under oil immersion (× 1000 magnification). A sufficient number of fields of vision were examined to enable up to 50 leukocytes to be inspected (no fewer than 200 fields of vision).

### Molecular characterization

Specific amplification of CNM and *Hepatozoon* spp. DNA was used for the identification of infections in all trapped voles (males and females), embryos and pups*.* The primers and thermal profiles used in this study have been described previously^44,45^. The PCR amplification of the 470 bp fragment of the *16S rRNA* with species-specific primers enabled the detection of CNM^1^. PCR amplification and sequencing of the 914 bp fragment of the heat-shock protein gene (*groEL* gene)^15,45^ were used for the detection and species identification of CNM. As positive controls, we used the genomic DNA of CNM extracted from the tick *I. ricinus*^15^.

The detection and genotyping of *Hepatozoon* spp. were performed by PCR amplification and sequencing of the 660 bp gene fragment of the *18S rRNA*, as described previously^39,44^. The DNA of *Hepatozoon erhardovae* from a bank vole^19^ was used as a positive control. Negative controls, consisting of sterile water, were included in each set of PCRs.

PCR products were subjected to electrophoresis on a 1.5% agarose gel, and stained with Midori Green stain (Nippon Genetics GmbH, Düren, Germany). Samples that tested positive on two consecutive occasions were considered to be positive. Selected positive products from the PCR reactions were subsequently sequenced (Genomed, Warsaw, Poland).

### Genotyping and phylogenetic analysis

Thirty six *Hepatozoon-*positive PCR products derived from 18 trapped voles, 16 products obtained from pups, and two products obtained from embryos were sequenced from both directions (Genomed, Warsaw, Poland).

Eighty three CNM-positive PCR products (50 for *16S rRNA* gene and 33 for *groEL* gene) from trapped voles and their offspring were sequenced (Genomed, Warsaw, Poland).

All the sequences were aligned using Molecular Evolutionary Genetics Analysis (MEGA) v. 11.0 open access software (https://www.megasoftware.net/). The evolutionary model was chosen according to the data and bootstrapped over 1000 randomly generated sample trees. The Maximum Likelihood method was used for tree-construction. Phylogenetic analyses encompassed the sequences obtained in the current study and sequences of *Hepatozoon* sp. and CNM deposited in the GenBank database^46^.

### Statistical analysis

The statistical approach adopted has been documented comprehensively in our earlier publications^42,43,47–50^. For the analysis of prevalence (percentage of animals infected) maximum likelihood techniques based on log-linear analysis of contingency tables (in SPSS vs 21) was applied. The results are presented as percentages with 95% confidence limits in parentheses (CL), calculated with bespoke software based on the tables of Rohlf and Sokal (1995), by courtesy of F.S. Gilbert and J. M. Behnke from the University of Nottingham, UK. For analysis of the prevalence of infections in wild-caught voles, we fitted prevalence of infection as a binary factor with host species (three levels: *M. arvalis, M. oeconomus, M. agrestis*), host sex (two levels: males and females), host age (three levels: juvenile, young adult, adult), and year (two levels: 2013, 2014) used as factors^42,43^. Subsequent analyses were carried out for each host species separately.

For analysis of the prevalence in pups, we implemented pup survival as a binary factor (dead = 0 or alive = 1 at the age of 3 weeks). In order to test the hypothesis that co-infection of *Hepatozoon* and CNM in females/dams may facilitate congenital transmission to their embryos/pups, we fitted models with CNM infection of female/dam and embryo/pup as an additional factor (coded as infected = 1, uninfected = 0). For each level of analysis in turn, beginning with the most complex model, involving all possible main effects and interactions, those combinations not contributing significantly to the explanation of variation in the data were eliminated stepwise, beginning with the highest-level interaction, as applied in our earlier papers^42,43^. A minimum sufficient model was then obtained, for which the likelihood ratio of χ^2^ was not significant, indicating that the model was sufficient in explaining the data. The success of vertical transmission to each litter, calculated as the fraction of positive pups/litter, was correlated with litter size using the Spearman rank correlation test (SPSS v. 21)^42,43^.

### Ethical statement

All of the procedures were conducted with the approval of the First Warsaw Local Ethics Committee for Animal Experimentation in Poland (ethical license numbers: 148/2011, 406/2013, and 517/2014) according to the principles governing experimental conditions and care of laboratory animals required by the European Union and the Polish Law on Animal Protection^42,43^. All animal care in the current study was conducted in accordance with ARRIVE (Animal Research: Reporting of In Vivo Experiments) guidelines 2.0^51^.

## Results

### Prevalence of *Hepatozoon* in the community of voles

In total, 217 voles of three species were trapped and sampled: 124 common voles, *M. arvalis*; 76 root voles, *M. oeconomus* and 17 field voles, *M. agrestis.* Prevalence of *Hepatozoon* sp. infection, based on PCR results by year of study, host species, and sex is provided in Table 1. In total, a positive product of the PCR reaction was obtained for 11.1% (95% CL: 8.5–14.3%) of voles in the community. The highest prevalence of *Hepatozoon* was found in *M. arvalis* (13.7% [95% CL: 9.1–19.8%]) and 9.2% [95% CL: 4.1–18.7%] of *M. oeconomus* tested positive, but no *Hepatozoon* infections were detected in *M.* *agrestis* (*Hepatozoon* infection × host species: *χ*^2^ = 5.60, *df* = 2, *P* = 0.06). Differences in prevalence of *Hepatozoon* between the two years of the study, between males and females (Table 1, NS), and between the three age classes were not significant (*Hepatozoon* infection × age class: *χ*^2^ = 2.47, *df* = 2, *P* = 0.29). Gamonts of *Hepatozoon* sp. were not observed in any of the inspected blood smears.

**Table 1:** Prevalence of *Hepatozoon* spp. in three species of wild-caught *Microtus* voles. Numbers of pregnant females and percentage (%) of pregnant infected females are shown in brackets.

Year		*M. arvalis*			*M. agrestis*			*M. oeconomus*			*Microtus* spp.		
	Infection	♂	♀	All	♂	♀	All	♂	♀	All	♂	♀	Total
2013	NI	20	29 (17)	49	9	5 (3)	14	10	6 (4)	16	39	40 (24)	79
	I	0	6 (3)	6	0	0 (0)	0	2	1 (1)	3	2	7 (4)	9
	% infected	0.0%	17.1% (15%)	10.9%	0.0%	0.0% (0%)	0.0%	16.7%	14.3% (20%)	15.8%	4.9%	14.9% (14%)	10.2%
2014	NI	29	29 (4)	58	1	2 (0)	3	23	30 (4)	53	53	61 (8)	114
	I	4	7 (6)	11	0	0 (0)	0	2	2 (1)	4	6	9 (7)	15
	% infected	12.1%	19.4% (60%)	15.9%	0.0%	0.0% (0%)	0.0%	8.0%	6.3% (20%)	7.0%	10.2%	12.9% (47%)	11.6%
∑	NI	49	58 (21)	107	10	7 (3)	17	33	36 (8)	69	92	101 (32)	193
	I	4	13 (9)	17	0	0 (0)	0	4	3 (2)	7	8	16 (11)	24
	% *Hepatozoon* infected	7.5%	18.3% (30%)	13.7%	0.0%	0.0% (0%)	0.0%	10.8%	7.7% (20%)	9.2%	8.0%	13.7% (26%)	11.1%

*NI* number of uninfected voles, *I* number of infected voles.

### Prevalence of infection in pregnant females and dams

Altogether 117 female voles were trapped, among which 43 were pregnant. Embryos were isolated from the uteri of thirty two gravid females. Embryos from 12 litters (including one litter from a *Hepatozoon*-positive *M. oeconomus* female) were too small to enable isolation (Fig. 1). Finally, 111 embryos from 20 female voles, including three *Hepatozoon*-positive *M. arvalis* were examined (Fig. 1, Table 2). Eleven dams were kept in captivity until 3 weeks after pup delivery (Fig. 1; host species and litter size are provided in Table 3).

**Table 2:** Evidence for vertical transmission of *Hepatozoon* sp. in embryos of female voles captured in 2013.

ID of pregnant female	Host species	No. of embryos in litter	No. of embryos infected with *Hepatozoon* in the litter	% of infected embryos
2013/45	*M. arvalis*	6	0	0.0%
2013/47	*M. arvalis*	2	0	0.0%
2013/72	*M. arvalis*	6	2	33.3%
∑	6 × *M. arvalis*	14	2	14.3%

**Table 3:** Evidence for vertical transmission of *Hepatozoon* sp. in pups delivered by female voles captured in 2014.

ID of pregnant female	Host species	No. of pups in litter	No. of pups infected with *Hepatozoon* in the litter	% of infected pups
2014/34	*M. arvalis*	5	2	40%
2014/59	*M. arvalis*	5	4	80%
2014/65	*M. arvalis*	6	4	67%
2014/77	*M. oeconomus*	6	3	50%
2014/126	*M. arvalis*	7	2	29%
2014/130	*M. arvalis*	4	3	75%
2014/131	*M. arvalis*	6	1	17%
∑	6 × *M. arvalis* 1 × *M. oeconomus*	39 33 *M. arvalis* + 6 *M. oeconomus*	19 16 *M. arvalis* + 3 *M. oeconomus*	48.7% (19/39) 48.5 (16/33) *M. arvalis*, 50.0% (3/6) *M. oeconomus*

The overall prevalence of *Hepatozoon* sp. infection in the pregnant females was 25.6% (95% CL: 13–42%). Prevalence was 30% (95% CL: 16.3–48.3%) in pregnant *M.* *arvalis* and 20% (95% CL: 3.7–55.4%) in *M.* *oeconomus* females (Table 1, Fig. 1).

### Detection of *Hepatozoon* in embryos (2013 and 2014)

*Hepatozoon* DNA was detected in two embryos obtained from one out of three *Hepatozoon*-positive *M. arvalis* females (14.3% (95% CL: 2.6–42.6%), Table 2). We did not detect *Hepatozoon* DNA in 97 embryos of the 17 *Hepatozoon*-negative females (Fig. 1).

### Detection of *Hepatozoon* in pups maintained under vector-free conditions (2014)

The DNA of *Hepatozoon* sp. was detected in pups from seven litters (6 *M.* *arvalis*, 1 *M.* *oeconomus*), however, none of the seven dams tested positive for *Hepatozoon.*

*Hepatozoon* sp. DNA was detected in 48.7% (95% CL: 33.9–63.2%) of pups (Fig. 1, Table 3). In one litter, from the *M.* *oeconomus* dam, 3 of 6 pups were positive (50% [95% CL: 15.3–84.7%]), in comparison to 48% (16/33 [95%CL: 33.9–63.2%]) of positive pups from six *M.* *arvalis* dams (Table 3) (NS).

No correlation was found between the percentage of *Hepatozoon*-positive pups in a litter and litter size (NS, Table 3). There was also no significant difference in the percentage of infected male and female pups born to infected dams: 52.6% (10/19 [95% CL: 31.2–74.3%]) of males and 45.0% (9/20 [95% CL: 24.4–68.0%]) of females were PCR-positive. There was no difference in body weight nor in survival of pups born with congenital infections, in comparison to the uninfected offspring of uninfected dams (mean body weight 15.63 + / − 2.8 g for infected and 15.74 + / − 2.23 g for uninfected pups).

### Genotyping of *Hepatozoon* sp

**Figure 3:**
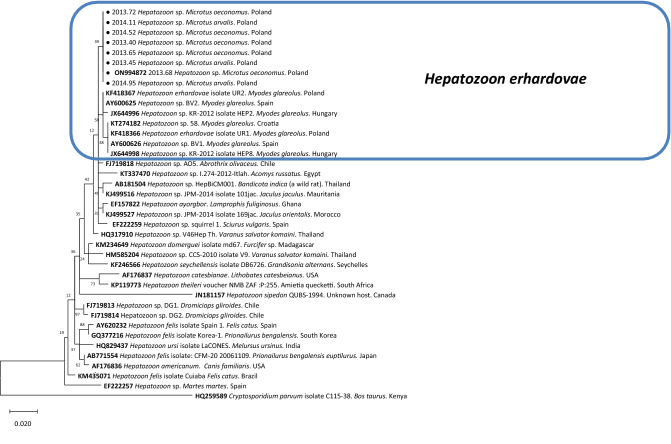
The phylogenetic tree of *Hepatozoon* based on a fragment of the *18S rRNA* gene, was inferred using the Maximum Likelihood method and a Tamura 3-parameter (I + G). The percentage of replicate trees in which the associated taxa clustered together in the bootstrap test (1000 replicates) are shown next to the branches. The analysis involved 38 nucleotide sequences. All positions containing gaps and missing data were eliminated. The nucleotide sequence of *Cryptosporidium parvum* was used as an outgroup. Evolutionary analyses were conducted in MEGA 11.0. Sequences obtained in the present study are marked with a black dot at the beginning.

In the phylogenetic tree inferred from the *18S rRNA* gene fragment (≈540 bp), our *Hepatozoon* sequences clustered within a large clade composed by many *Hepatozoon* genotypes associated with rodents and reptiles from different parts of the world (Fig. 3). This clade was sister to another large clade that contained *Hepatozoon* sequences associated with canids (i.e. *H. canis*) and felids (*Hepatozoon felis*). The topology of the tree supported the closest similarity of *Hepatozoon* sp. from *Microtus* spp. to *Hepatozoon erhardovae*, originating from bank voles (*M. glareolus*) from the same location in NE Poland^21^. However, sequences from *Microtus* voles differed slightly from both main conserved genotypes of *H. erhardovae* in bank voles across Europe^52^ and formed a separate branch. Sequences of *Hepatozoon* obtained in a mother and in the offspring were identical. Representative sequence have been deposited in GenBank under accession number ON994872.

### Prevalence of CNM in the community of voles

Prevalence of CNM infection by year of study, host species and sex is provided in Table 4. In total, a positive product of the PCR reaction was obtained for 35.5% (95% CL: 31.2–39.9%) of *Microtus* voles in the community. The highest prevalence of CNM was detected in *M. agrestis* (47.1% [95% CL: 25.3–71.3%]) and the lowest in *M. oeconomus* (23.7% [95% CL: 14.7–35.4%]) but the difference in prevalence between the three host species was not significant (NS).

**Table 4:** Prevalence of “*Ca.* Neoehrlichia mikurensis*“* in three species of wild-caught *Microtus* voles. Numbers of pregnant females and percentage (%) of pregnant infected females are shown in brackets.

Year		*M. arvalis*			*M. agrestis*			*M. oeconomus*			*Microtus* spp.		
	Infection	♂	♀	All	♂	♀	All	♂	♀	All	♂	♀	Total
2013	NI	8	18 (12)	26	5	2 (1)	7	8	4 (3)	12	21	24 (16)	45
	I	12	17 (8)	29	4	3 (2)	7	4	3 (2)	7	20	23 (12)	43
	% infected	60.0%	48.6% (40%)	52.7%	44.4%	60.0% (66%)	50.0%	33.3%	42.9% (40%)	36.8%	48.8%	48.9% (42.9%)	48.9%
2014	NI	21	26 (5)	47	1	1 (0)	2	19	27 (4)	46	41	54 (9)	95
	I	12	10 (5)	22	0	1 (0)	1	6	5 (1)	11	18	16 (6)	34
	% infected	36.4%	27.8% (50%)	31.9%	0.0%	50.0%	33.3%	24.0%	15.6% (20%)	19.3%	30.5%	22.9% (40%)	26.4%
∑	NI	29	44 (17)	73	6	3 (1)	9	27	31 (7)	58	62	78 (25)	140
	I	24	27 (13)	51	4	4 (2)	8	10	8 (3)	18	38	39 (18)	77
	% CNM-infected	45.3%	38.0 (43.3%)	41.1%	40.0%	57.1 (66%)	47.1%	27.0%	20.5% (30%)	23.7%	38.0%	33.3% (41.9%)	35.5%

*NI* number of uninfected voles, *I* number of infected voles.

**Figure 4:**
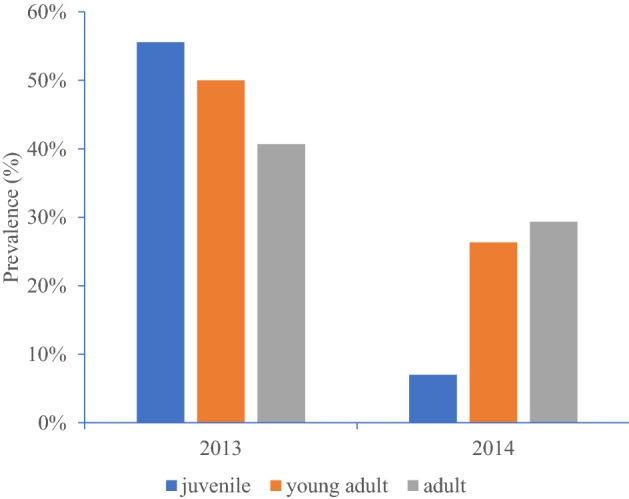
Prevalence of “*Ca.* Neoehrlichia mikurensis” in three age classes of wild-caught voles sampled in 2013–2014.

Overall prevalence was almost twice as high in voles captured in 2013 compared to those sampled in 2014 (*χ*^2^ = 9.05, *df* = 1, *P* < 0.05). There was a significant interaction of year of study and host age and prevalence of CNM (age class x CNM infection × year of study: *χ*^2^ = 11.05, *df* = 2, *P* = 0.004). In 2013 prevalence declined gradually with increasing host age but in 2014, a year with generally low prevalence, this pattern was reversed (Fig. 4). Prevalence was similar in males and females (no significant association; Table 4).

### CNM infection in females and dams

One hundred and eleven embryos from 20 necropsied females were examined. Among the females, five *M. arvalis* females, one *M. oeconomus* and two *M. agrestis* female tested positive for CNM (Table 5).

**Table 5:** Evidence for vertical transmission of “*Ca*. Neoehrlichia mikurensis” in embryos extracted from pregnant female voles.

ID of female	Host species	No. of infected /total embryos in litter	% of embryos infected with CNM
2013/3	*M. arvalis*	2/7	28.6%
2013/15	*M. arvalis*	1/4	25.0%
2013/20	*M. arvalis*	0/5	0.0%
2013/45	*M. arvalis*	0/6	0.0%
2013/52	*M. arvalis*	0/6	0.0%
2013/7	*M. agrestis*	0/4	0.0%
2013/24	*M. agrestis*	0/7	0.0%
2013/70	*M. oeconomus*	0/6	0.0%
∑	8 × *Microtus* sp.: 5 × *M. arvalis* 2 × *M. agrestis* 1 × *M. oeconomus*	3/45 *Microtus* sp.: 3/28 *M. arvalis* + 0/11 *M. agrestis* + 0/6 *M. oeconomus*	6.6% *Microtus* sp.: 10.7% *M. arvalis* + 0.0*% M. agrestis* + 0.0% *M. oeconomus*

Among eleven dams kept in captivity until pup delivery, 5 *M. arvalis* and 1 *M. oeconomus* females tested positive for CNM (host species and litter size provided in Table 6).

**Table 6:** Evidence for vertical transmission of “*Ca*. Neoehrlichia mikurensis” in pups delivered by female voles captured in 2014.

ID of dam	Host species	No. of infected /total pups in litter	% of pups infected with CNM
2014/25	*M. arvalis*	0/6	0%
2014/34	*M. arvalis*	2/5	40%
2014/59	*M. arvalis*	0/5	0%
2014/65	*M. arvalis*	3/6	50%
2014/112	*M. arvalis*	0/5	0%
2014/77	*M. oeconomus*	2/6	33%
∑	6 × *Microtus* sp.: 5 × *M. arvalis* 1 × *M. oeconomus*	7/33 *Microtus* sp.: 5/27 *M. arvalis* + 2/6 *M. oeconomus*	21.2% *Microtus* sp.: 18.5% *M. arvalis* + 33.3% *M. oeconomus*

The overall prevalence of CNM infection in the pregnant females was 41.9% (95% CL: 26.2–58.8%) (Table 4), ranging 30–66% among pregnant females of the three host species (Fig. 2, Table 4).

### Detection of CNM in embryos (2013 and 2014)

The DNA of CNM was detected in 12 euthanised pregnant females. Infection in four litter (3 *M. arvalis,* 1 *M. oeconomus*), could not be evaluated because of the early stage of pregnancy, with embryos too small to enable reliable isolation of fetal tissues.

The DNA of CNM was detected in embryos from two out of five litters from CNM-positive *M. arvalis* females (Table 5). No CNM DNA was detected among 17 embryos obtained from CNM-positive *M. oeconomus* and *M. agrestis* females, nor in embryos from the CNM-negative females (Table 5, Fig. 2).

### Detection of CNM in pups maintained under vector-free conditions (2014)

The DNA of CNM was detected in pups from two out of five litters from infected *M. arvalis* dams and in one litter from an infected *M. oeconomus* dam. In total, CNM DNA was detected in 21.2% (95% CL: 11.2–35.7%) of pups (Fig. 2, Table 6). Prevalence of congenital CNM infection was similar (19–33%) in *M. arvalis* and *M. oeconomus* pups (NS, Table 6).

There was no correlation between the percentage of CNM-positive pups in a litter and litter size (NS, Table 6). No impact of CNM infection on pup survival, nor on body weight, was observed (mean body weight 14.92 g + / − 3.13 for infected and 15.82 g + / − 2.31 for uninfected pups).

All the pups born to CNM infected dams survived in comparison to two litters of non-infected dams that died after delivery (1 *M. oeconomus,* 1 *M. arvalis*). There was also no significant difference in the percentage of congenital infections between male and female pups born to infected dams: 16% (3/19) of males and 29% (4/14) of females were CNM-positive.

### Genotyping of CNM

Thirty three sequences of the CNM *groEL* gene fragment were obtained from PCR-positive trapped voles and their offspring, representing three host species (24 from *M. arvalis*, 6 from *M. agrestis* and 3 from *M. oeconomus*). There was almost no diversity among the obtained sequences displaying 99.8–100% identity to CNM ‘MgUR’ isolate (KJ561570) derived from a bank vole, *M. glareolus,* from the same area in our earlier study (Welc-Falęciak et al., unpublished). A representative *groEL* sequence has been deposited in GenBank under accession number OP158204 (Suppl. File 1).

Fifty sequences of the CNM *16S rRNA* gene fragment were obtained from PCR-positive trapped voles and their offspring, representing all three host species (34 from *M. arvalis*, 5 from *M. agrestis,* and 11 from *M. oeconomus*). There was no diversity among the obtained sequences displaying 100% identity to CNM WAW5 isolate (KJ123754) derived from a asymptomatic patient in Warsaw^17^, but also to isolate Omsk-41_Micagr (MN736126) derived from *M.* *agrestis* in Syberia (Rar et al., unpublished). A representative *16S rRNA* sequence has been deposited in GenBank under accession number (OQ152532).

### Co-infection of *Hepatozoon* sp. and CNM in dams and pups

Co-infections of *Hepatozoon* and CNM were detected in four dams (vole ref nos. 2014/34, 2014/59, 2014/65, and 2014/77). The vertical transmission of both *Hepatozoon* and CNM had occurred in three out of those litters. Another two dams (2014/25 and 2014/112) were infected with CNM but not with *Hepatozoon* and in this case congenital CNM infection was not detected in pups. Vertical transmission of *Hepatozoon* occurred in three litters of dams infected only with *Hepatozoon* but not with CNM (vole ref nos. 2014/126, 2014/130 and 2014/131).

In a minimal sufficient model obtained from this analysis, only *Hepatozoon* infection in a dam was associated with *Hepatozoon* infection in pups (*χ*^2^ = 9.54, *df* = 5, *P* < 0.05). CNM infection in a dam was associated with CNM infection in pups (*χ*^2^ = 9.18, *df* = 2, *P* < 0.05). *Hepatozoon* infection in a dam was not associated with congenital infection of CNM, while CNM infection in a dam was not associated with congenital infection of *Hepatozoon* in pups (NS).

Furthermore, focusing on the infection status of offspring, we correlated the success of vertical transmission of *Hepatozoon* in a litter (percentage of the litter with *Hepatozoon*) with the success of vertical transmission of CNM in the litter (percentage of litter with CNM), for offspring of co-infected females/dams (n = 4) but no correlation was evident (NS).

## Discussion

In the present study, we have reported on the relatively high prevalence of infection with the zoonotic bacterium CNM in a sympatric *Microtus* vole community inhabiting a rural area in North-Eastern Poland. Moreover, we have provided further evidence that this high prevalence is likely to have been maintained by a significant rate of congenital infections (vertical transmission from naturally infected female voles to their offspring). Our study is among the first to assess the prevalence of *Hepatozoon* sp. and to determine the genetic identity of this pathogen in a *Microtus* spp. community, providing support also for the possibility of vertical transmission of *Hepatozoon* among vole species.

We have identified CNM in three species of *Microtus* voles. Although CNM has been previously reported in *M. arvalis* and *M. agrestis*^5,10,53^, this is the first report of CNM in *M. oeconomus*. Thus, we have expanded the list of rodent species serving as reservoirs of these zoonotic bacteria. Our study has confirmed that rodents are the main reservoir hosts for CNM because no CNM infections have been detected previously in insectivores^10,53^ and other *Neoehrlichia* species have been found only in carnivores^54^.

The prevalence of CNM in our vole community ranged 24–47% depending on *Microtus* species. This is a moderate rate of prevalence, similar values having been reported in at least seven papers for the most commonly studied rodent species in several countries in Europe^5,8,10,13,53,55,56^. In one study, much lower values (prevalence < 2%) were reported^7^ and in two studies in Germany prevalence (> 55%) was found to be slightly higher compared to that in our work^9,12^. Hence, there is a some disparity in prevalence values for the most commonly studied species, ranging 0.3–33% for *A. agrarius*^7,12,57^, 1.7–65% for *A. flavicollis*^5,7–10,12,13,53,56,57^, 1.1–58% for *M. glareolus*^5,7–10,12,13,53,55–57^, and 11–33% for *A. sylvaticus*^5,8,10,53,56^.

*Microtus* spp. have been less well studied than mice and bank voles, with reported CNM prevalences of 5–30% for *M. arvalis*^5,10,13^ and 8% for *M. agrestis*^53^. The prevalence value reported in this study (overall 35.5% in *Microtus* spp.) is in agreement with these studies. Importantly, high prevalence of zoonotic CNM in *Microtus* spp. may be of greater significance than high prevalence in *Apodemus* spp. or *M. glareolus,* because *Microtus* spp. voles can live in close proximity to humans, inhabiting any kind of open areas (abandoned areas, field margins, gardens, petrol stations, grassy forecourts, etc.) *Microtus* spp. populations can reach high densities, thus constituting an important wildlife reservoir of infection for ticks and humans. Low genetic diversity of these bacteria^2,3^, derived from human cases, ticks and rodents, supports a significant role of rodents as the source of infection for humans. Interestingly, in two previous studies carried out in Poland, similar high prevalences of CNM were detected in *M. glareolus* (18–30%) in the same region of the country (North-Eastern Poland)^57^, in two murine species in Warsaw (23% in *A. flavicollis* and 11% in *A. agrarius*) and in 24–50% of rodents (27–29% *A. agrarius*, 29–36% *A. flavicollis*, 24–50% *M. glareolus*) from South-Western Poland, near Wrocław^18,57^.

The present study was planned to investigate the phenomenon of vertical transmission of vector-borne pathogens, bacteria and protists in naturally-infected rodent populations. In our previous papers we documented the occurrence of vertical transmission for *Bartonella* spp.^43^ and *Babesia microti*^42^ among three species of voles. In the present study we have extended this route of transmission to other pathogens, verifying that vertical transmission is also a key feature of infections with CNM and *Hepatozoon* sp. The DNA of CNM was detected in embryos and pups from infected *Microtus* females. In total, the DNA of CNM was detected in 21% of pups born to CNM infected dams and in 7.3% of embryos obtained from infected female voles. Thus, we have confirmed the occurrence of vertical transmission in two *Microtus* spp. Our findings support the previous report of vertical transmission of CNM to embryos and neonates of three rodent species from Germany^10^ and the discovery of a CNM-positive foetus in a litter of an *A. flavicollis* female from Slovakia^7^.

Moreover, the successful detection of vertical transmission of CNM in our study supports results presented by Obiegała et al. (2014)^10^: congenitally infected offspring were identified in 60% (9 out of 15 ) of litters, with a CNM prevalence of 34% (23 out of 67 individuals) in rodent foetuses and neonates from positive dams. Among those 15 litters, congenitally infected offspring were found in: 7/12 litters of *M. glareolus*, 1/1 litter of *A. flavicollis,* and 1/2 litters of *M. arvalis*^10^.

In the present study we observed also a declining prevalence of CNM infection with increasing age in the free-living voles sampled in 2013. A similar pattern has been described for prevalence of *Bartonella* in this vole community^43^. The highest prevalence in the youngest voles is consistent with vertical transmission of CNM in vole populations. Furthermore, as stated earlier, a prevalence of CNM in rodents up to 10 times higher than in tick populations supports the existence of transmission routes other than tick-borne and confirms the key role of rodents as reservoir hosts^10,15,16^.

Infections of *Hepatozoon* spp. have been reported in at least nine rodent species in Europe^9,20,21,40,58–63^ but by far the majority of studies concern *M. glareolus* (as the main host of *Hepatozoon*) and other host species have been studied rarely. Also among the studied species, prevalence of *Hepatoozoon* sp./*H. erhardowae* has been reported to be highest in *M. glareolus* in Europe, ranging 17–88%^9,20,21,40,61–64^. In our long-term study on haemoparasites in Masuria, North-Eastern Poland^21^, prevalence of *H. erhardovae* oscillated in the range 40–70% in bank voles during an 11 year period. Interestingly, two main genotypes of *H. erhardovae* (BV1 and BV2) seem to be highly conserved and distributed across distant regions of Europe- Spain, South Germany, Poland^21,23,52^.

Prevalence of *Hepatozoon* is generally lower in *Apodemus* spp., ranging 5–28% for *A. flavicollis* and 18–30% for *A. sylavaticus*^9,61,63^. There are few studies of *Hepatoozoon* in *Microtus* spp. voles, and prevalence, determined solely based on microscopy, has been reported mostly as zero^62,64^ or as a single positive individual^40^. Prevalence of *Hepatoozoon* sp./*H. lavieri,* with identification based on molecular techniques, has ranged 3–9% in *M. arvalis*^41,61^; 2–10% in *M. agrestis*^61,62^ and 7% in *M. oeconomus* from North-Eastern Poland^40^. In the current study, we detected *Hepatozoon* infection only in two species of voles, in *M. arvalis* (14%) and *M. oeconomus* (9%), but with slightly higher prevalence than reported previously.

In our study all the *Hepatoozoon* sequences that we obtained were very similar (95–100% homology), and based on the topology of the phylogenetic tree, they are likely to constitute either a *Microtus-*adapted variant/genotype of *H. erhardovae* or, less likely, a different species (*H. lavieri*?^25^). However, based on *18S rRNA* there is apparently little diversity among *Hepatozoon* isolates obtained from various rodents, amphibians and reptiles^24,26^.

One of the main findings of the present study is confirmation of vertical transmission of *Hepatozoon* in rodents by the detection of DNA in embryos and pups. Success of vertical transmission was high for pups, close to 50% both in six litters of *M. arvalis* and in one litter of *M. oeconomus*. However, no *Hepatozoon* infection was detected in dams. The lack of detection of *Hepatozoon* in dams may have been due to the low burden of parasites, confirmed also by a failure to detect *Hepatozoon* gamonts by microscopy in PCR-positive animals. Low burdens of parasites are typical for chronic infections and it may be pertinent that we have recently described successful vertical transmission of *B. microti* from chronically infected BALB/c mice to their offspring, while no such transmission occurred during the acute phase of infection^65,66^.

More than 60 years ago, vertical transmission of *Hepatozoon griseisciuri* was described in naturally-infected grey squirrels kept until partitution in a laboratory, under ectoparasite-free conditions^67^. Prevalence of infection was 92% in free-living grey squirrels and different life stages of *Hepatozoon* were then observed in 19 out of 21 pups (90%) (36 h to 4 weeks in age) but no *Hepatozoon* stages were detected in histological sections of different organs of a single two-week-old embryo^67^. Interestingly, similar high success of vertical transmission has been observed for *H. canis* in Beagle dogs (23/29 [79%] of infected pups in six litters from 3 infected bitches^35^) and in red foxes (2/3 positive foetuses [67%] from an infected vixen^37^). Thus, vertical transmission appears to be an established route of transmission for these vector-borne parasites in different host species and our study of *Hepatozoon* in rodents is consistent with this idea. Vertical transmission is likely to significantly contribute to the maintenance and spread of *Hepatozoon* spp., even in areas where competent vectors do not occur. Further investigation is needed to examine the viability of the agents found in the offspring, and the exact route of transmission (tranplacental, trans-uterine, through birth canal, etc.).

## Conclusions

The high prevalence of CNM infection in our *Microtus* spp. community may be the result of a relatively high rate of vertical transmission of CNM in three species of naturally infected voles. Vertical transmission was demonstrated also for *Hepatozoon* sp. in *M. arvalis* and *M. oeconomus*. Our study underlines the significance of alternative routes of transmission of important vector-borne pathogens.

## Supplementary Information

Supplementary Information.

## Data Availability

All relevant data are included in the article. Representative sequence of *Hepatozoon* sp. have been deposited in GenBank under accession number ON994872 (https://www.ncbi.nlm.nih.gov/nuccore/ON994872.1?report=GenBank). A representative sequences of CNM were deposited in GenBank under accession numbers OP158204 (https://www.ncbi.nlm.nih.gov/nuccore/OP158204.1?report=GenBank), and OQ152532 (https://www.ncbi.nlm.nih.gov/nuccore/OQ152532).
