# Supplementary material for: Candidatus Neoehrlichia mikurensis and Hepatozoon sp. in voles (Microtus spp.): occurrence and evidence for vertical transmission
Source: Sci Rep. 2023 Jan 31;13:1733. doi: 10.1038/s41598-023-28346-0 (PMC9889374; doi:10.1038/s41598-023-28346-0)
Supplement: Supplementary file 1 — Supplementary Information. [file 41598_2023_28346_MOESM1_ESM.pdf]

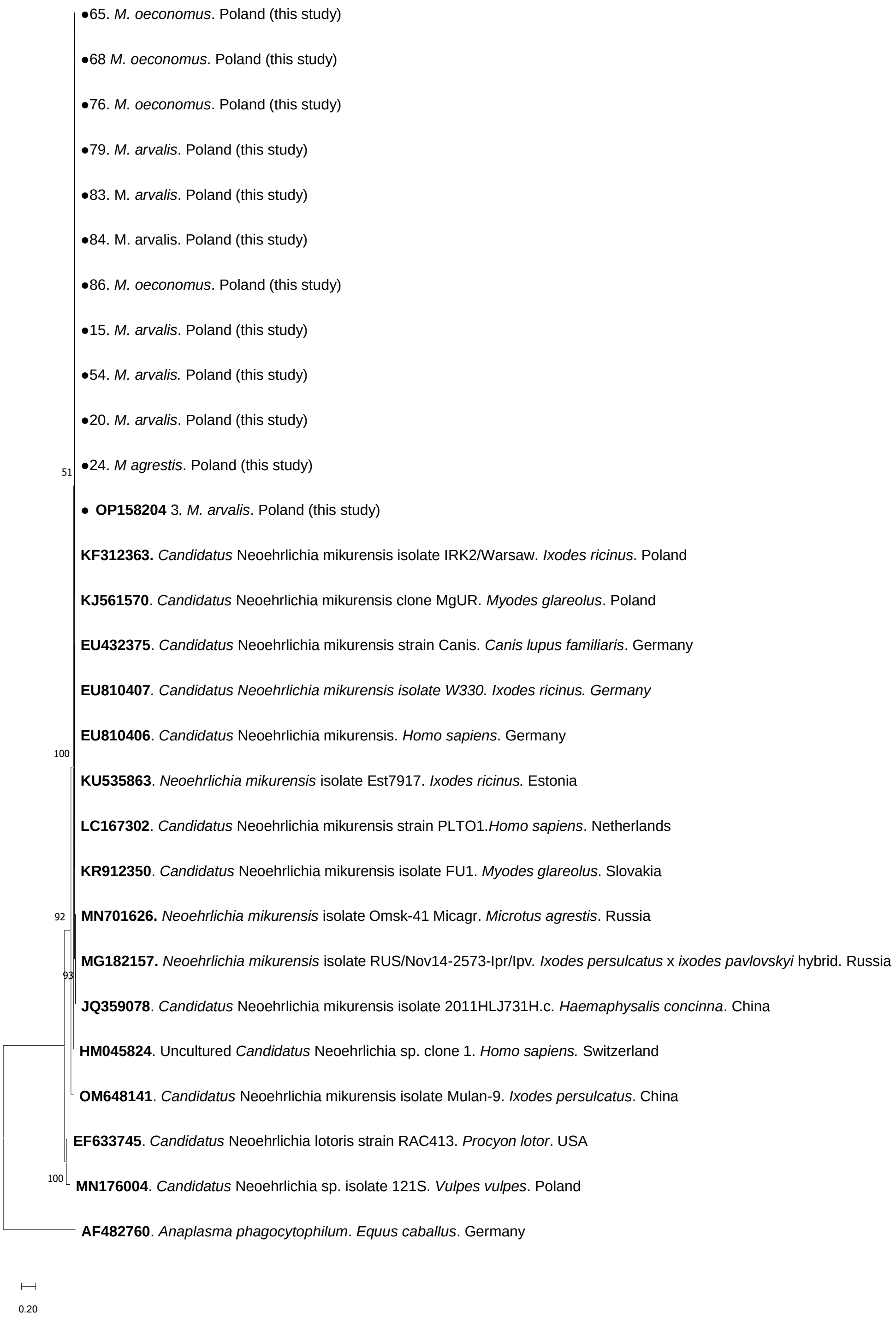

Suppl. File. 1 The phylogenetic tree of *Ca.* Neoehrlichia mikurensis based on a fragment of the *groEL* gene, was inferred using the Maximum Likelihood method and a Tamura 3-parameter (I + G). The percentage of replicate trees in which the associated taxa clustered together in the bootstrap test (1000 replicates) are shown next to the branches. The analysis involved 28 nucleotide sequences. All positions containing gaps and missing data were eliminated. The nucleotide sequence of *Anaplasma phagocytophilum* was used as an outgroup. Evolutionary analyses were conducted in MEGA 11.0. Sequences obtained in the present study are marked with a black dot at the beginning.
